# The Association of Dietary Vitamin Intake Time Across a Day With Cardiovascular Disease and All-Cause Mortality

**DOI:** 10.3389/fcvm.2022.822209

**Published:** 2022-03-23

**Authors:** Wenbo Gu, Huanyu Wu, Cong Hu, Jiaxu Xu, Hongyan Jiang, Yujia Long, Tianshu Han, Xue Yang, Wei Wei, Wenbo Jiang

**Affiliations:** The National Key Discipline, Department of Nutrition and Food Hygiene, School of Public Health, Harbin Medical University, Harbin, China

**Keywords:** chrono-nutrition, dietary vitamins, intake time, cardiovascular disease mortality, all-cause mortality

## Abstract

**Background:**

Chrono-nutrition emphasized the importance of the intake time; however, less is known about the impact of dietary vitamin intake time on health. This study aimed to examine our hypothesis about which vitamin intake time could influence the natural course of cardiovascular disease (CVD).

**Methods:**

A total of 27,455 adults enrolled in the National Health and Nutrition Examination Survey (NHANES) during 2003–2014 were recruited. The 12 dietary vitamin intakes in the morning, afternoon, and evening were categorized into tertiles or quartiles. Cox-proportional hazard regression models were developed to evaluate the association of vitamin intake time with CVD and all-cause mortalities.

**Results:**

Compared with participants in the lowest quartile, participants in the highest quartile of dietary VB2 intake in the morning had significantly lowest mortality risk of CVD [hazard ratio (HR)_VB2_ = 0.75, 95% CI: 0.60–0.94, *p* = 0.017]; whereas, participants in the highest quartile of dietary-vitamin B6 (VB6), vitamin C (VC), vitamin E (VE), and folate-equivalent consumed in the evening showed the lowest risks of CVD (HR_VB6_ = 0.77, 95% CI: 0.60–0.99, *p* = 0.103; HR_VC_ = 0.80, 95% CI: 0.65–0.98, *p* = 0.050; HR_VE_ = 0.75, 95% CI: 0.56–0.99, *p* = 0.032; HR_folate–equivalent_ = 0.78, 95% CI: 0.63–0.97, *p* = 0.116) and all-cause mortalities (HR_VB6_ = 0.81, 95% CI: 0.71–0.93, *p* = 0.006; HR_VC_ = 0.85, 95% CI: 0.76–0.95, *p* = 0.004; HR_VE_ = 0.84, 95% CI: 0.72–0.97, *p* = 0.011; HR_folate–equivalent_ = 0.80, 95% CI: 0.71–0.90, *p* = 0.001). Moreover, equivalently replacing 10% intake of dietary VB6, VC, VE, and folate-equivalent in the morning with evening were associated with 4% (HR_VB6_ = 0.96, 95% CI: 0.92–0.99), 5% (HR_VC_ = 0.95, 95% CI: 0.92–0.99), 4% (HR_VE_ = 0.96, 95% CI: 0.91–0.99), and 5% (HR_folate–equivalent_ = 0.95, 95% CI: 0.92–0.99) lower risk of CVD mortality.

**Conclusion:**

This study found that the optimal intake time of dietary VB2 was in the morning, and the optimal intake times of dietary VB6, VC, VE, and folate-equivalent were in the evening.

## Introduction

Chrono-nutrition is a new nutritional research field, which aims to explore whether and how the intake time of diet would affect health (1). Accumulating studies have demonstrated that diet intake time is important for wellbeing, independent of the quality and quantity of a diet (2, 3). Currently, most of the studies have mainly focused on the intake time of energy and macronutrients and have demonstrated that consuming them at different time periods influenced the oscillation of the circadian clock genes, resulting in different health effects (4, 5); however, few studies have examined whether and how the micronutrient intake time would impact health.

Vitamins, as important components of micronutrients, play important roles in maintaining the healthy physiological functions of an organism. A huge number of epidemiological studies have reported the association between the nutritional status of dietary vitamins and cardiovascular disease (CVD); however, epidemiological research and randomized controlled trial (RCT) experiment results are inconsistent (6–8). Previous animal studies have demonstrated that dietary vitamins regulated the oscillation of circadian clock genes in the central nervous system and were involved in the synthesis of the circadian rhythm neurotransmitters (9, 10). The disrupted clock genes and circadian rhythm neurotransmitters have been demonstrated to be related to the development of CVD (11, 12). Based on this evidence, this study hypothesized that the intake time could influence the natural course of CVD. To examine our hypothesis, this study assessed the relationship between the dietary vitamins consumed at different time periods across a day and all-cause and CVD mortalities in the US population from the National Health and Nutrition Examination Survey (NHANES).

## Materials and Methods

### Study Population

The NHANES was launched by the National Center for Health Statistics (NCHS), covering the samples of the non-institutionalized civilian population in the United States. The information of NHANES was described elsewhere (13). In this study, participants from 2003 to 2014 who met the following criteria were selected: adults (aged ≥18 years), who were not missing information of diet and mortality, who were skipping breakfast, and those whose total energy intake ranged from 450 to 5,000 kcal/d. A total of 27,455 subjects, including 13,359 men and 14,096 women, were included in this study. The institutional review board approval of the NCHS was obtained. All subjects provided written informed consent.

### Dietary Assessment

The detailed dietary consumption for 2 non-consecutive days was collected *via* 24-h dietary recall interviews. The first 24-h dietary recall was conducted through an in-person interview; after 3–10 days, the second 24-h dietary recall was further conducted over the phone. Dietary nutrients and energy consumption were calculated based on the U.S. Department of Agriculture’s Food and Nutrient Database for Dietary Studies. The use of dietary supplements was obtained using the questionnaire. According to the MyPyramid Equivalent Database 2.0 (MPED 2.0) of the U.S. Department of Agriculture’s surveyed food, the nutrient intakes of 37 MyPyramid main food categories were calculated.

### Main Exposure

The amounts of 12 dietary vitamin intakes at different time periods across a day were the main exposures of this study, including vitamin A (VA), vitamin B1 (VB1), vitamin B2 (VB2), vitamin B6 (VB6), vitamin B12 (VB12), vitamin C (VC), vitamin D (VD), vitamin E (VE), vitamin K (VK), retinol (RET), niacin, and folate-equivalent. The consumption of vitamins in the morning, afternoon, and evening were calculated based on the gross of breakfast and snack before lunch (< 12:00), lunch and snack after lunch (12:00–18:00), dinner and snack after dinner (> 18:00), respectively.

### Main Outcomes

The variable of outcome was the status of mortality as determined by the National Death Index (NDI) until December 31, 2015. The NDI is a considerably reliable and widely used death identification resource. The ICD-10 was used to determine disease-specific death. ICD-10 codes I00–I09, I11, I13, I20–I51, or I60–I69 were assigned to death due to CVD. During the 178,228 person-years of follow-up, a total of 2,680 deaths were included for further analysis, with 805 deaths due to CVD.

### Covariates

In this study, non-dietary covariates included age, sex, race/ethnicity, body mass index (BMI, kg/m^2^), annual household income, smoking status (defined as smoking in the past 5 days), drinking status (defined as drinking at least 12 times in the past year), education level, regular exercise (defined as having moderate to high-intensity physical exercise in the past month), diseases of hypertension (defined by a self-reported diagnosis, the systolic blood pressure ≥ 90 mmHg, or the diastolic blood pressure ≥ 140 mmHg), hyperlipidemia (defined by a self-reported diagnosis, serum triglyceride ≥ 2.26 mmol/L, or serum cholesterol ≥ 6.22 mmol/L, or low-density lipoprotein ≥ 4.14 mmol/L), diabetes (defined by a self-reported diagnosis, HbA1c level ≥ 6.5%, or a fasting plasma glucose level ≥ 7.0 mmol/L), medicine control for cholesterol, serum glucose, and blood pressure, as well as a history of CVD, stroke, and cancer (all the above variables are defined based on previous physician diagnoses). Dietary quality was measured based on the Alternative Healthy Eating Index (AHEI) (14), dietary nutritional supplement use (defined as using any dietary supplements in the previous 30 days), total energy intake per kg, and total dietary vitamin intake. Except for the age, BMI, and dietary information, all the other variables are categorical variables.

### Statistical Analysis

Demographic characteristics, dietary nutrient intakes, and anthropometrics were presented using the mean of continuous variables and the percentage of categorical variables. The general linear model adjusted for age, sex, and energy intake per kg were utilized to compare baseline characteristics. The cumulative risk curves were drawn to describe the CVD and all-cause mortality risks of participants who were in different vitamin intake quartiles at optimal time periods. The statistical analyses were conducted using the R 4.0.2 software, and *P* < 0.05 on both sides was regarded to be statistically significant.

### Cox Proportional Hazard Models

The intakes of dietary vitamins except for VD in the morning, afternoon, and evening were categorized into quartiles. Dietary VD consumption at each period could be only divided into tertiles. Cox proportional hazard (CPH) models were used to estimate hazard ratios (HR) and 95% CI associated with all-cause and CVD mortalities for vitamin consumption over time. The timescale in the Cox model used follow-up time that was calculated using person-months from the interview date to death, or December 31, 2015, whichever came first. A series of potential confounders were controlled, including age; sex; race; smoking; drinking; BMI, exercise; education; income; hypertension; hyperlipidemia; diabetes; medicine control for cholesterol, serum glucose, and blood pressure; history of cancer, stroke, and CVD; total dietary vitamin intake; the diet quality of AHEI; total daily energy intake per kg; and dietary nutritional supplement use.

### Predicted Equivalent Vitamin Substitution Models

On the basis of CPH models developed earlier, this study further built equivalently vitamin substitution models to assess the changes in CVD mortality by switching one vitamin intake at a single time point to another. A key rationale of this analysis is that the reduction in vitamins provided by one time period will be replaced by the same vitamins provided by another time period when intake of total vitamins and other nutrients are held constant. In this study, we equivalently switched 10% of dietary VB2 intake from the afternoon or evening to morning and 10% of dietary VB6, VC, VE, and folate-equivalent intake from the morning or afternoon to the evening, respectively, to examine whether and how the mortality risk of CVD would change.

### Sensitivity Analysis

In this study, we performed three sets of sensitivity analyses to test the robustness of our results. In the first set, we analyzed the relationship between total dietary vitamin intake and all-cause and CVD mortalities to examine whether the vitamin consumption time would provide more information than the total vitamin intake. In the second and third sets, we tested whether sex and the status of hypertension, hyperlipidemia, and diabetes, as well as a history of CVD, stroke, and cancer, would modify the association of vitamin intake time with all-cause and CVD mortalities. CPH models were further performed to analyze the modified effects of stroke and CVD for the association between CVD mortality and intake time of several specific vitamins.

## Results

### Baseline Characteristics

The demographic and nutritional characteristics of the participants are presented in Table 1. Compared with survived people, the participants who died due to CVD and all-cause mortalities were more likely to be men; to be older; to have had a higher prevalence of hypertension, diabetes, hyperlipidemia; and to have had a history of stroke, CVD, and cancer; to be more prone to taking medicine for cholesterol, blood pressure, and serum glucose; to have more dietary intake of VA, VB1, VB2, VB6, VC, VE, VK, niacin, and folate-equivalent; to have a less dietary intake of energy; and to have lower smoking rate, drinking rate, education level, annual income, BMI, and AHEI (all the *P* < 0.05).

**TABLE 1:** Baseline characteristics of variables in survived-people, CVD mortality, and all-cause mortality status.

Variables	Survival-people (*N* = 24,775)	CVD mortality (*N* = 805)	*P*-value (Survival people vs. CVD mortality)	All-cause mortality (*N* = 2,680)	*P*-value (Survival people vs. all-cause mortality)
Age (years)	47.0 (18.0)	72.4 (11.7)	0.001	69.8 (14.5)	0.001
Male (%)	47.7	58.8	0.001	57.5	0.001
Non-Hispanic white (%)	45.4	63.0	0.081	61.3	0.001
Current smoking (%)	22.9	17.8	0.001	20.3	0.001
Current drinking (%)	64.4	58.9	0.001	59.6	0.001
College graduate or above (%)	51.0	32.3	0.001	34.3	0.001
>$100,000 annual household income (%)	11.8	1.7	0.001	2.5	0.001
BMI (kg/m^2^)	28.7 (6.7)	28.6 (6.3)	0.001	28.2 (6.4)	0.016
Regular exercise (%)	23.9	19.1	0.242	19.3	0.541
Dietary nutritional supplement use (%)	48.3	60.7	0.474	58.5	0.187
Hypertension (%)	61.5	76.8	0.001	70.2	0.007
Diabetes (%)	13.9	35.2	0.042	31.0	0.011
Hyperlipidemia (%)	30.9	47.2	0.001	43.2	0.001
Stroke (%)	2.7	16.0	0.001	14.0	0.001
Cardiovascular disease (%)	3.2	18.6	0.001	14.1	0.001
Cancer (%)	8.0	21.7	0.001	23.5	0.001
Medicine for cholesterol (%)	23.1	42.5	0.001	37.7	0.001
Medicine for blood pressure (%)	24.7	60.6	0.001	52.8	0.006
Medicine for serum glucose (%)	23.7	41.9	0.001	38.2	0.001
AHEI	53.14 (13.50)	47.59 (12.39)	0.001	48.66 (12.86)	0.001
Total energy intake (kcal/d)	2073.581 (798.09)	1683.58 (606.37)	0.001	1767.57 (682.14)	0.001
Total vitamin A intake (mcg/d)	621.02 (541.09)	636.29 (575.37)	0.008	658.63 (686.78)	0.026
Total vitamin B1 intake (mg/d)	1.62 (0.78)	1.44(0.67)	0.001	1.47 (0.68)	0.001
Total vitamin B2 intake (mg/d)	2.11 (1.06)	1.94 (0.88)	0.001	2.02 (0.96)	0.001
Total vitamin B6 intake (mg/d)	2.06 (1.25)	1.75 (0.89)	0.001	1.80 (0.99)	0.001
Total vitamin B12 intake (mcg/d)	5.18 (5.13)	4.96 (5.43)	0.308	5.26 (6.92)	0.568
Total vitamin C intake (mg/d)	89.72 (82.09)	85.18 (71.87)	0.147	84.36 (71.84)	0.003
Total vitamin D intake (mcg/d)	3.48 (4.45)	1.97 (3.34)	0.001	2.25 (3.65)	0.001
Total vitamin E intake (mg/d)	7.68 (5.09)	5.97 (3.56)	0.001	6.31 (3.99)	0.001
Total vitamin K intake (mcg/d)	105.02 (175.33)	89.60 (123.16)	0.001	94.74 (135.86)	0.001
Total niacin intake (mg/d)	25.11 (12.47)	20.14 (8.81)	0.001	20.88 (9.88)	0.001
Total folate equivalent intake (mcg/d)	538.17 (309.41)	473.38 (259.06)	0.001	484.53 (268.15)	0.001
Total RET intake (mcg/d)	418.68 (429.43)	447.02 (514.67)	0.671	461.24 (625.55)	0.058

*Continuous variables are presented as mean (standard deviation). Categorical variables are presented as a percentage. Adjustments included age, sex, and total dietary energy intake per kg.*

### Cox Proportional Hazard Models

**FIGURE 1:**
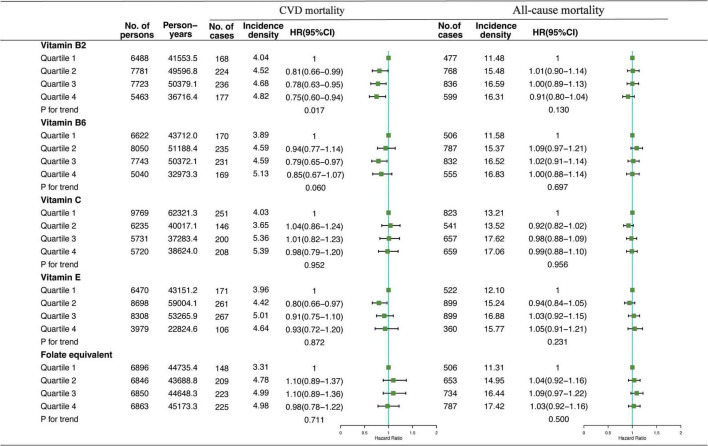
Multivariate adjusted hazard ratios (HRs) of the dietary vitamin B2 (VB2), vitamin B6 (VB6), vitamin C (VC), vitamin E (VE), and folate-equivalent intake in the morning with cardiovascular disease (CVD) and all-cause mortalities. Adjustments included age, sex, ethnicity, income, education level, regular exercise, smoking and drinking status, and BMI; prevalence of diabetes, hypertension, and hyperlipidemia; history of cancer, stroke, and cardiovascular disease; medicine control for cholesterol, serum glucose, and blood pressure; and dietary nutritional supplement use, Alternative Healthy Eating Index (AHEI), total daily energy intake per kg, and total dietary vitamin intake. Incidence density (per1,000 person-years); Q, quartile.

**FIGURE 2:**
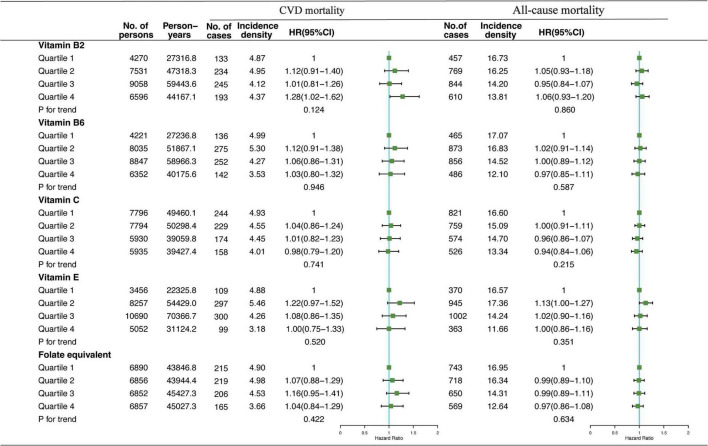
Multivariate adjusted HRs of the dietary-VB2, VB6, VC, VE, and folate-equivalent intake in the afternoon with CVD and all-cause mortalities. Adjustments included age, sex, ethnicity, income, education level, regular exercise, smoking and drinking status, and BMI; prevalence of diabetes, hypertension, hyperlipidemia; history of cancer, stroke, and cardiovascular disease; medicine control for cholesterol, serum glucose, and blood pressure; and dietary nutritional supplement use, AHEI, total daily energy intake per kg, and total dietary vitamin intake.

**FIGURE 3:**
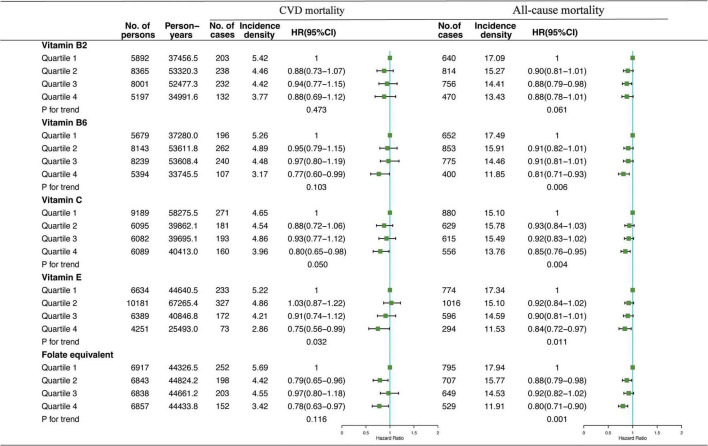
Multivariate adjusted HRs of the dietary-VB2, VB6, VC, VE, and folate-equivalent intake in the evening with CVD and all-cause mortalities. Adjustments included age, sex, ethnicity, income, education level, regular exercise, smoking and drinking status, and BMI; prevalence of diabetes, hypertension, and hyperlipidemia; history of cancer, stroke, and cardiovascular disease; medicine control for cholesterol, serum glucose, and blood pressure; and dietary nutritional supplement use, AHEI, total daily energy intake per kg, and total dietary vitamin intake.

Association of VB2, VB6, VC, VE, and folate-equivalent intakes in the morning, afternoon, and evening with all-cause and CVD mortalities is presented in Figures 1–3. Association of other kinds of vitamin intakes in the morning, afternoon, and evening with all-cause and CVD mortalities is presented in Supplementary Tables 1–3. In the morning (Figure 1), as indicated by HR and 95% CI, the participants in the highest quartile (Q4) of dietary VB2 intake had the lowest risk of CVD mortality (HR_VB2_ = 0.75, 95% CI: 0.60–0.94, *p* = 0.017) than those in the lowest quartile (Q1). In the afternoon (Figure 2), no significant association of 12 vitamin intakes with all-cause and CVD mortalities was observed. In the evening (Figure 3), participants who were in the highest intake quartiles (Q4) of VB6, VC, VE, and folate-equivalent intakes had the lowest CVD mortality (HR_VB6_ = 0.77, 95% CI: 0.60–0.99, *p* = 0.103; HR_VC_ = 0.80, 95% CI: 0.65–0.98, *p* = 0.050; HR_VE_ = 0.75, 95% CI: 0.56–0.99, *p* = 0.032; HR_folate–equivalent_ = 0.78, 95% CI: 0.63–0.97, *p* = 0.116) and all-cause mortality (HR_VB6_ = 0.81, 95% CI: 0.71–0.93, *p* = 0.006; HR_VC_ = 0.85, 95% CI: 0.76–0.95, *p* = 0.004; HR_VE_ = 0.84, 95% CI: 0.72–0.97, *p* = 0.011; HR_folate–equivalent_ = 0.80, 95% CI: 0.71–0.90, *p* = 0.001) than those in the lowest quartiles (Q1). The cumulative risk curves of CVD and all-cause mortality risks of participants in Q1–Q4 at their optimal intake times are presented in Supplementary Figure 1.

### Equal-Vitamin Intake Substitution Analysis

**FIGURE 4:**
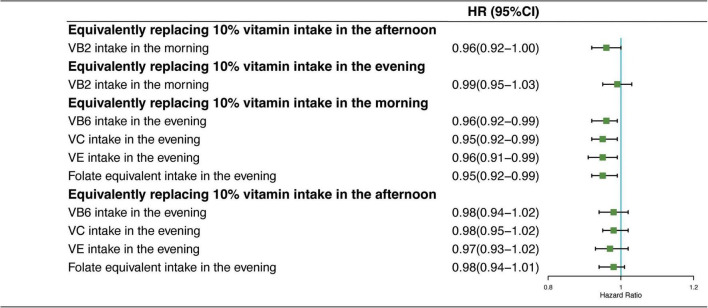
Multivariate adjusted HRs of the dietary vitamin intake with CVD mortality: equivalent dietary-VB2, VB6, VC, VE, and folate-equivalent substitution from afternoon and evening to morning. Adjustments included age, sex, ethnicity, income, education level, regular exercise, smoking and drinking status, and BMI; prevalence of diabetes, hypertension, and hyperlipidemia; history of cancer, stroke, and cardiovascular disease; medicine control for cholesterol, serum glucose, and blood pressure; and dietary nutritional supplement use, AHEI, total daily energy intake per kg, and total dietary vitamin intake.

In Figure 4, the results showed the mortality risk changes in CVD in two sets of predicted models: (1) equivalently switching VB2 intake from afternoon or evening to morning and (2) equivalently switching VB6, VC, VE, and folate-equivalent intake from morning or afternoon to evening. The HR of CVD mortality showed no significant reduction in CVD mortality due to the intake time substitution of equivalent dietary VB2; and equivalent substitution intake of VB6, VC, VE, and folate-equivalent in the afternoon to that in the evening had no significant effects. In contrast, HR of CVD mortality decreased by 4% (HR_VB6_ = 0.96, 95% CI: 0.92–0.99), 5% (HR_VC_ = 0.95, 95% CI: 0.92–0.99), 4% (HR_VE_ = 0.96, 95% CI: 0.91–0.99), and 5% (HR_folate–equivalent_ = 0.95, 95% CI: 0.92–0.99), respectively, when 10% of VB6, VC, VE, and folate-equivalent consumed in the morning was equivalently switched to the evening.

### Sensitivity Analysis

In the first set of sensitivity analyses, the total intake of each dietary VB2, VB6, and VC was not associated with CVD mortality in the two models, suggesting that analysis of their intake time provided more information about CVD mortality than the amounts of total daily intake. A high total intake of dietary VE was associated with reduced mortality risk of CVD in the two models, which implied both amounts of VE intake and intake time of VE were associated with the CVD mortality (Supplementary Table 4). Besides, in model 1, the participants in the highest quartile (Q4) of dietary folate-equivalent intake had a lower mortality risk of CVD than the participants in the lowest quartile (Q1); however, we observed no significant protective effect of CVD mortality in the model 2, suggesting that folate-equivalent intake time was likely to be the underlying mechanism of the causation of folate-equivalent and CVD mortality. Therefore, results highlighted that vitamin intake time played an important role in the protection of CVD mortality. The second set showed that sex did not modify the association of dietary VB2, VB6, VC, and folate-equivalent consumption time with CVD mortality, and only women with higher VE intake in the evening showed lower mortality risk for CVD (Supplementary Tables 5, 6). The third set showed that the status of hypertension, hyperlipidemia, diabetes, and cancer did not modify the association of vitamin consumption time with CVD mortality, except for the status of stroke (VB2, VB6, and folate-equivalent in the morning and VB2 and VB6 in the evening) and CVD (VB2 and VB6 in the evening). However, for patients with stroke and CVD, no significant association of the abovementioned vitamin intake at different times with CVD mortality was observed (Supplementary Tables 7–10). These results implied that the protective effect of vitamin consumption time on CVD mortality should be further examined in participants with specific diseases.

## Discussion

Based on the U.S. NHAHES from 2003 to 2014, this study examined the association of 12 dietary vitamin intakes at different time periods across a day with all-cause and CVD mortalities. Among these vitamins, a high intake of dietary VB2 in the morning was associated with a low mortality risk of CVD, and high intakes of dietary VB6, VC, VE, and folate-equivalent in the evening were associated with lower all-cause and CVD mortalities. Moreover, replacing 10% intakes of dietary VB6, VC, VE, and folate-equivalent in the morning with that in the evening reduced CVD mortality by 4, 4, 4, and 5% when their total daily dietary intakes were held constant.

Although the association between dietary vitamins and CVD has been widely reported, the conclusions of epidemiological studies and RCTs are inconsistent (6–8, 15). Accumulating studies have shown the effect of chrono-nutrition on human beings; however, it has been completely ignored in previous studies, which were only based on the overall intake quantity of vitamins, due to limitations of the vitamin intake data in each meal. For the first time, we interpreted the relationship between vitamin intake and CVD and all-cause mortalities from the perspective of vitamin intake timing. This study found that, among 12 dietary vitamins, the intake time modified the association of dietary VB2, VB6, VC, and folate-equivalent with all-cause and CVD mortalities, independent of other traditional dietary risk factors, in particular, overall diet quality and amounts of total dietary vitamin intake. Moreover, this study has also found that both the amount and intake time of VE were associated with all-cause and CVD mortalities. In addition, these results prompted us to identify the significance of consumption time of these dietary vitamins for improving the long-term survival of CVD.

In our study, we observed that a higher intake of VB2 in the morning was associated with a lower mortality risk of CVD. On one hand, the circadian rhythm of cryptochrome 2 (Cry2) is likely the possible mechanism that underpins this observation. Flavin adenine dinucleotide (FAD) is a type of VB2, which not only stimulates the synthesis of Cry2 but also maintains the stability of Cry2 expression (16). As a FAD-based blue-light photoreceptor, Cry2 regulates the circadian clock in mammals (17). In healthy individuals, it has been reported that the daily pattern of Cry2 expression level shows the greatest peak in the morning (18). The increased intake of dietary VB2 in the morning is more compatible with the peak of Cry2 for holding the healthy rhythm of cardiovascular activity, inflammation, oxidative stress, vascular endothelium repair process, and glycolipid metabolism (19), which further reduces the CVD mortality. On the other hand, VB2 also inhibits platelet aggregation, improves myocardial function and ischemia, and reduces myocardial infarction (20–22). Evidence documented that platelet activity, blood pressure, and the incidence rate of CVD, including myocardial infarction, were high at dawn and morning (23). Therefore, increased VB2 intake in the morning probably counteracts these harmful activities contributing to CVD, which is another possible mechanism of VB2 intake in the morning to improve cardiovascular health. In the substitution analysis, when replacing the dietary VB2 intake in the evening with morning, no reduction in the risk of CVD mortality was observed, indicating that VB2 might assist other vitamins to promote cardiovascular health in the evening, such as VB6 and VC (24–26).

Moreover, we also found that increased intake of dietary VB6 and folate-equivalent in the evening could result in reduced CVD mortality. So far, the RCTs have not confirmed that the vitamin B group (especially VB6 and folic acid) could reduce the risk of CVD mortality (27, 28); however, our study has surprisingly found that they could affect CVD mortality *via* the intake time. Higher intakes of VB6 and folate-equivalent in the evening likely had a more potentially beneficial effect on CVD mortality, which could be supported by previous studies. First, the synthesis of 5hydroxytryptamine (5-HT) and melatonin is activated in the evening. VB6 is an important vitamin that promotes the generation of 5-HT (29), and the folate-equivalent is also well-known to provide the methyl that participates in the last step of the melatonin synthesis (30). Therefore, higher VB6 and folate-equivalent consumption in the evening may enhance the levels of 5-HT and melatonin. Previous studies have also reported that 5-HT and melatonin were important factors in regulating circadian rhythm, sleep-wake cycles, oxidative stress rhythm, and cardiovascular health (31, 32). Second, as homocysteine is an outstanding risk factor of CVD (33), and its peak period is usually at night, the high intake of VB6 and folate-equivalent in the evening may therefore more effectively reduce the level of homocysteine by accelerating its metabolic process and then present their beneficial effects on reducing the CVD mortality (34, 35). Third, animal studies have found that dietary folate intake might downregulate the expression of the circadian gene, such as the Cry1 and Per2 *via* DNA methylation (36, 37). In human tissues, these genes showed the lowest mRNA levels during the midnight to daybreak (38, 39). Therefore, a high intake of folate-equivalent in the evening probably argument the oscillation of the circadian clock genes, which is likely to show more adherence to the natural circadian pattern of these clock genes.

The antioxidant and anti-inflammation capabilities of VC and VE have been documented in previous animal studies (40, 41), which result in a growing interest in assessing whether dietary VC and VE intakes might help to prevent CVD; however, the results based on the human studies were less consistent (6, 42). It has been documented that oxidative stress and inflammation-related genes have circadian rhythms, which could maintain blood pressure and regulate cardiovascular system function (43, 44). Some major antioxidants (GSH-PX, MDA, SOD, and melatonin) and white blood cells (lymphocytes, neutrophils, and monocytes) of humans were reported to peak at night or before daybreak (45). Based on our results, increased VC and VE intake in the evening could reduce the mortality risk of CVD, probably because higher intake of VC and VE in the evening were likely more compatible with the circadian pattern of antioxidants and inflammation. Furthermore, serum cholesterol frequently peaks in the evening, and animal studies have demonstrated that VC and VE could reduce endogenous cholesterol and prevent atherosclerosis through hydroxylation metabolism and inhibiting the synthesis of endogenous cholesterol, respectively (46, 47). Therefore, more VC and VE intakes in the evening were probably more effective for these physiological effects.

### Strengths and Limitations

This study has several strengths. First, it was the first one to investigate whether and how the intake time modified the association of vitamins with all-cause and CVD mortalities on the basis of the high-quality data from NHANES, adding new knowledge for the field of chrono-nutrition. Second, the association documented in our study was considerably robust, which could be independent of a suite of classical dietary confounders, particularly, overall diet quality, dietary supplements use, and total dietary vitamin intake. However, we also recognized that our study had some certain limitations. First, although the self-reported 24-h dietary recall is the most common tool for obtaining dietary information in observational studies, people consumed numerous types and amounts of food each day, so there will be certain measurement errors in recalling. Second, the NHANES database used two dietary measurements within 2 weeks to predict the survival of the entire population, but it is worth considering that individual eating habits might change over time. Third, although various potential confounders were controlled in this observational study, some unmeasured confounding factors still cannot be removed.

## Implication and Conclusion

Nutrition is an important part to maintain public health. Our results were integrated into the nutritional guidelines and intervention strategies. The optimal consumption time of dietary VB2 for reducing the mortality risk of CVD is likely in the morning, and the optimal consumption time of VB6, VC, folate-equivalent, and VE for reducing the mortality risk of CVD is likely in the evening. This information is of importance in providing nutritional recommendations for the public.

In a word, the consumption time impacted the association of dietary VB2, VB6, VC, and folate-equivalent with CVD mortality. Therefore, we advised consuming more dietary VB2 in the morning and dietary VB6, VC, VE, and folate-equivalent in the evening for reducing the risk of CVD mortality.

## Data Availability Statement

The original contributions presented in the study are included in the article/Supplementary Material, further inquiries can be directed to the corresponding author/s.

## Ethics Statement

The studies involving human participants were reviewed and approved by National Center for Health Statistics Research Ethics Review Board. The patients/participants provided their written informed consent to participate in this study.

## Author Contributions

TH, WJ, and WW conceived the study design. WG, HW, JX, and HJ did the statistical analysis. YL and CH repeated and validated the statistical analysis. WW, TH, and WJ wrote the manuscript and attests that all listed authors meet authorship criteria and that no others meeting the criteria have been omitted. TH was the guarantor. CH and XY revised the manuscript. All authors provided critical revisions of the draft and approved the submitted draft.

## Conflict of Interest

The authors declare that the research was conducted in the absence of any commercial or financial relationships that could be construed as a potential conflict of interest.

## Publisher’s Note

All claims expressed in this article are solely those of the authors and do not necessarily represent those of their affiliated organizations, or those of the publisher, the editors and the reviewers. Any product that may be evaluated in this article, or claim that may be made by its manufacturer, is not guaranteed or endorsed by the publisher.

## Abbreviations

AHEI: alternative healthy eating index
BMI: body mass index
CVD: cardiovascular disease
CPH: Cox proportional hazards
Cry2: cryptochrome 2
FAD: flavin adenine dinucleotide
CI: confidence interval
HR: hazard ratios
NCHS: National Center for Health Statistics
NDI: National Death Index
NHANES: National Health and Nutrition Examination Survey
Q: quartile
RCTs: randomized controlled trials
RET: retinol
VA: vitamin A
VB1: vitamin B1
VB2: vitamin B2
VB6: vitamin B6
VB12: vitamin B12
VC: vitamin C
VD: vitamin D
VE: vitamin E
VK: vitamin K
5-HT: 5hydroxytryptamine.
