# Supplementary material for: The Association of Dietary Vitamin Intake Time Across a Day With Cardiovascular Disease and All-Cause Mortality
Source: Front Cardiovasc Med. 2022 Mar 23;9:822209. doi: 10.3389/fcvm.2022.822209 (PMC8984283; doi:10.3389/fcvm.2022.822209)
Supplement: Supplementary file 1 [file Data_Sheet_1.pdf]

Figure Legend

Supplementary figure 1. The cumulative risk curves of CVD and all-cause mortality risks of participants in Quarter1-Quarter4 at their optimal intake-times.

(a) VB2 intake in the morning; (b) VB6 intake in the evening; (c) VC intake in the evening; (d) VE intake in the evening; (e) Folate equivalent intake in the evening.

Supplementary table 1. Multivariate adjusted HRs of the dietary vitamin intake in themorning with all-cause and CVD mortality.

Vitamins	CVD Mortality					All-cause Mortality		
	No. of persons	Person-years	No. of cases	Incidence density	HR (95% CI)	No. of cases	Incidence density	HR (95% CI)
**Vitamin A**								
Quartile 1	6901	44989.5	162	3.60	1	525	11.67	1
Quartile 2	6839	44499.7	212	4.76	0.99(0.80-1.21)	670	15.06	1.00(0.89-1.12)
Quartile 3	6864	44985.6	212	4.71	0.87(0.70-1.07)	719	15.98	0.96(0.86-1.08)
Quartile 4	6851	43770.9	219	5.00	0.84(0.67-1.04)	766	17.50	0.96(0.86-1.08)
P for trend					0.046			0.387
**Vitamin B1**								
Quartile 1	5865	38057.4	124	3.26	1	417	10.96	1
Quartile 2	8736	55544.8	265	4.77	1.05(0.85-1.30)	849	15.29	1.04(0.93-1.18)
Quartile 3	7327	47731.8	238	4.99	1.02(0.81-1.27)	793	16.61	1.05(0.93-1.19)
Quartile 4	5527	36911.7	178	4.82	0.92(0.72-1.17)	621	16.82	0.99(0.87-1.13)
P for trend					0.390			0.797
**Vitamin B12**								
Quartile 1	3843	25221.9	88	3.49	1	292	11.58	1
Quartile 2	8534	54212.7	241	4.45	1.00(0.78-1.28)	747	13.78	0.96(0.84-1.10)
Quartile 3	9480	61757.7	308	4.99	0.95(0.75-1.21)	1032	16.71	1.01(0.89-1.15)
Quartile 4	5598	37053.5	168	4.53	0.79(0.61-1.04)	609	16.44	0.91(0.78-1.05)
P for trend					0.052			0.327
**Vitamin D**								
Quartile 1	10233	92413.9	490	5.30	1	1495	16.18	1
Quartile 2	1362	6986.2	17	2.43	0.63(0.36-1.09)	75	10.74	0.91(0.69-1.19)
Quartile 3	15860	78845.6	298	3.78	0.73(0.53-1.01)	1110	14.08	0.93(0.78-1.11)
P for trend					0.067			0.310
**RET**								
Quartile 1	6890	45003.8	156	3.46	1	506	11.24	1
Quartile 2	6847	44606.0	203	4.55	0.94(0.76-1.16)	674	15.11	1.02(0.91-1.14)
Quartile 3	6865	44755.4	221	4.94	0.93(0.75-1.15)	719	16.07	0.99(0.88-1.11)
Quartile 4	6853	43850.5	225	5.13	0.87(0.70-1.09)	781	17.81	0.99(0.87-1.11)
P for trend					0.218			0.653

**Vitamin K**								
Quartile 1	12268	82310.5	343	4.17	1	148	13.95	1
Quartile 2	6351	41891.6	218	5.20	1.03(0.87-1.23)	684	16.33	0.99(0.90-1.08)
Quartile 3	4403	28142.6	141	5.01	1.01(0.83-1.24)	460	16.35	0.98(0.88-1.10)
Quartile 4	4433	25901.0	103	3.98	0.89(0.71-1.12)	388	14.98	0.99(0.88-1.12)
P for trend					0.450			0.816
**Niacin**								
Quartile 1	7176	46981.1	169	3.60	1	564	12.00	1
Quartile 2	7512	48496.6	263	5.42	1.07(0.88-1.30)	779	16.06	1.01(0.90-1.12)
Quartile 3	6749	43854.4	202	4.61	0.88(0.72-1.09)	713	16.27	0.99(0.88-1.10)
Quartile 4	6018	38913.6	171	4.39	0.82(0.66-1.03)	624	16.04	0.96(0.85-1.08)
P for trend					0.020			0.444

Adjustments included age, sex, ethnicity, income, education level, regular exercise, smoking and drinking status, BMI; prevalence of diabetes, hypertension, hyperlipidemia; history of cancer, stroke, cardiovascular disease; medicine control for cholesterol, serum glucose, and blood pressure; dietary nutrient supplement use, AHEI, total daily energy intake per kg and total dietary vitamin intake.

Supplementary table 2. Multivariate adjusted HRs of the dietary vitamin intake in the afternoon with all-cause and CVD mortality.

**Vitamins**	**CVD Mortality**					**All-cause Mortality**		
	**No. of persons**	**Person-years**	**No. of cases**	**Incidence density**	**HR (95% CI)**	**No. of cases**	**Incidence density**	**HR (95% CI)**
**Vitamin A**								
Quartile 1	6898	43835.0	216	4.93	1	729	16.63	1
Quartile 2	6832	45312.3	195	4.30	1.00(0.82-1.22)	625	13.79	0.92(0.83-1.02)
Quartile 3	6871	44759.2	183	4.09	0.98(0.80-1.20)	660	14.75	1.00(0.89-1.11)
Quartile 4	6854	44339.2	211	4.76	1.15(0.94-1.41)	666	15.02	1.02(0.91-1.14)
P for trend					0.243			0.511
**Vitamin B1**								
Quartile 1	4059	26057.6	124	4.76	1	447	17.15	1
Quartile 2	7140	45631.4	234	5.13	1.15(0.92-1.44)	761	16.68	1.02(0.90-1.15)
Quartile 3	9445	61683.3	285	4.62	1.21(0.98-1.51)	911	14.77	1.02(0.90-1.14)
Quartile 4	6811	44873.5	162	3.61	1.09(0.85-1.40)	561	12.50	0.96(0.84-1.09)
P for trend					0.440			0.503
**Vitamin B12**								
Quartile 1	4652	29397.8	141	4.80	1	491	16.70	1
Quartile 2	4626	29647.2	129	4.35	0.99(0.78-1.26)	457	15.41	0.99(0.87-1.12)
Quartile 3	10752	69802.8	331	4.74	1.15(0.94-1.41)	1052	15.07	1.01(0.90-1.12)
Quartile 4	7425	49397.9	204	4.13	1.15(0.92-1.44)	680	13.77	1.03(0.91-1.16)
P for trend					0.092			0.562
**Vitamin D**								
Quartile 1	13212	107060.9	555	5.18	1	1742	16.27	1
Quartile 2	3226	16232.7	53	3.27	0.92(0.66-1.28)	222	13.68	1.07(0.91-1.26)
Quartile 3	11017	54952.1	197	3.58	1.02(0.80-1.29)	716	13.03	1.02(0.90-1.16)
P for trend					0.914			0.996
**RET**								
Quartile 1	6955	43711.5	210	4.80	1	729	16.68	1
Quartile 2	6794	44963.1	190	4.23	1.01(0.83-1.24)	612	13.61	0.92(0.83-1.03)
Quartile 3	6862	45145.7	192	4.25	1.05(0.86-1.28)	649	14.38	0.97(0.87-1.09)
Quartile 4	6844	44425.5	213	4.79	1.18(0.96-1.44)	690	15.53	1.04(0.93-1.16)

P for trend					0.113			0.373
**Vitamin K**								
Quartile 1	8711	56732.1	292	5.15	1	965	17.01	1
Quartile 2	6591	44149.7	212	4.80	1.07(0.90-1.28)	662	14.99	0.98(0.89-1.08)
Quartile 3	6081	40081.4	171	4.27	1.00(0.83-1.22)	562	14.02	0.95(0.86-1.06)
Quartile 4	6072	37282.4	130	3.49	0.94(0.76-1.18)	491	13.17	0.99(0.89-1.12)
P for trend					0.658			0.672
**Niacin**								
Quartile 1	6824	43664.0	213	4.88	1	722	16.54	1
Quartile 2	7397	48183.4	263	5.46	1.16(0.97-1.39)	863	17.70	1.09(0.99-1.21)
Quartile 3	6869	45612.8	198	4.34	1.09(0.89-1.33)	619	13.57	0.97(0.87-1.09)
Quartile 4	6365	40785.4	131	3.21	0.98(0.78-1.23)	486	11.92	1.01(0.89-1.14)
P for trend					0.919			0.633

Adjustments included age, sex, ethnicity, income, education level, regular exercise, smoking and drinking status, BMI; prevalence of diabetes, hypertension, hyperlipidemia; history of cancer, stroke, cardiovascular disease; medicine control for cholesterol, serum glucose, and blood pressure; dietary nutrient supplement use, AHEI, total daily energy intake per kg and total dietary vitamin intake.

Supplementary table 3. Multivariate adjusted HRs of the dietary vitamin intake in the evening with all-cause and CVD mortality.

Vitamins	CVD Mortality					All-cause Mortality		
	No. of persons	Person-years	No. of cases	Incidence density	HR (95% CI)	No. of cases	Incidence density	HR (95% CI)
**Vitamin A**								
Quartile 1	6864	44701.4	208	4.65	1	670	14.99	1
Quartile 2	6884	44569.9	204	4.58	0.91(0.75-1.11)	677	15.19	0.93(0.83-1.03)
Quartile 3	6865	45068.9	180	3.99	0.82(0.67-1.01)	658	14.60	0.89(0.80-0.99)
Quartile 4	6842	43905.5	213	4.85	0.88(0.71-1.08)	675	15.37	0.84(0.75-0.95)
P for trend					0.149			0.004
**Vitamin B1**								
Quartile 1	5427	35016.8	180	5.14	1	615	17.56	1
Quartile 2	8395	54379.0	273	5.02	1.06(0.87-1.28)	855	15.72	0.94(0.84-1.04)
Quartile 3	8517	55288.3	238	4.30	1.00(0.82-1.23)	793	14.34	0.91(0.82-1.02)
Quartile 4	5116	33561.6	114	3.40	0.91(0.71-1.17)	417	12.42	0.87(0.76-1.00)
P for trend					0.407			0.041
**Vitamin B12**								
Quartile 1	4503	28963.0	138	4.76	1	457	15.78	1
Quartile 2	8966	57745.7	278	4.81	1.08(0.88-1.33)	878	15.20	0.99(0.89-1.11)
Quartile 3	9148	59339.5	269	4.53	1.04(0.84-1.29)	913	15.39	1.00(0.89-1.13)
Quartile 4	4838	32197.5	120	3.73	0.94(0.73-1.23)	432	13.42	0.94(0.81-1.08)
P for trend					0.646			0.513
**Vitamin D**								
Quartile 1	9867	90273.1	493	5.46	1	1521	16.85	1
Quartile 2	3292	16288.4	58	3.56	0.88(0.61-1.28)	192	11.79	0.79(0.64-0.96)
Quartile 3	14296	71684.3	254	3.54	0.77(0.57-1.05)	967	13.49	0.80(0.68-0.95)
P for trend					0.045			0.022
**RET**								
Quartile 1	6877	43843.8	195	4.45	1	677	15.44	1
Quartile 2	6893	44906.8	226	5.03	1.14(0.94-1.38)	664	14.79	0.95(0.85-1.05)
Quartile 3	6850	45046.1	193	4.28	0.93(0.76-1.14)	696	15.45	0.93(0.83-1.04)

Quartile 4	6835	44449.1	191	4.30	0.96(0.77-1.19)	643	14.47	0.89(0.79-0.99)
P for trend					0.338			0.058
**Vitamin K**								
Quartile 1	8286	55658.4	275	4.94	1	913	16.40	1
Quartile 2	6390	42556.9	181	4.25	0.94(0.77-1.13)	623	14.64	0.94(0.85-1.04)
Quartile 3	6392	40736.5	181	4.44	0.92(0.75-1.12)	595	14.61	0.88(0.79-0.98)
Quartile 4	6387	39293.9	168	4.28	0.92(0.74-1.14)	549	13.97	0.85(0.75-0.95)
P for trend					0.391			0.002
**Niacin**								
Quartile 1	7178	47125.6	246	5.22	1	843	17.89	1
Quartile 2	7182	46844.7	245	5.23	1.12(0.94-1.35)	729	15.56	0.94(0.85-1.04)
Quartile 3	6546	42115.1	179	4.25	1.03(0.84-1.25)	610	14.48	0.98(0.88-1.09)
Quartile 4	6549	42160.4	135	3.20	0.87(0.69-1.09)	498	11.81	0.87(0.78-0.99)
P for trend					0.215			0.057

Adjustments included age, sex, ethnicity, income, education level, regular exercise, smoking and drinking status, BMI; prevalence of diabetes, hypertension, hyperlipidemia; history of cancer, stroke, cardiovascular disease; medicine control for cholesterol, serum glucose, and blood pressure; dietary nutrient supplement use, AHEI, total daily energy intake per kg and total dietary vitamin intake.

Supplementary table 4. Multivariate adjusted HRs of the total dietary vitamin intake with all-cause and CVD mortality.

			Model1						Model2					
			CVD Mortality			All-cause Mortality			CVD Mortality			All-cause Mortality		
Vitamins	No. of persons	Person-years	No. of cases	Incidence density	HR (95% CI)	No. of cases	Incidence density	HR (95% CI)	No. of cases	Incidence density	HR (95% CI)	No. of cases	Incidence density	HR (95% CI)
**Vitamin B2**														
Quartile 1	6599	41454.4	219	5.28	1	683	16.48	1	219	5.28	1	683	16.48	1
Quartile 2	9001	57546.3	278	4.83	1.00(0.84-1.20)	894	15.54	1.00(0.90-1.11)	278	4.83	1.07(0.88-1.30)	894	15.54	1.03(0.93-1.15)
Quartile 3	6885	45296.5	201	4.44	0.94(0.73-1.16)	690	15.23	0.99(0.88-1.11)	201	4.44	1.01(0.80-1.27)	690	15.23	1.04(0.92-1.18)
Quartile 4	4970	33942.1	107	3.15	0.91(0.70-1.17)	413	12.17	1.03(0.90-1.18)	107	3.15	0.99(0.74-1.32)	413	12.17	1.11(0.95-1.29)
P for trend					0.387			0.791			0.868			0.241
**Vitamin B6**														
Quartile 1	6542	43574.7	259	5.94	1	839	19.25	1	259	5.94	1	839	19.25	1
Quartile 2	9352	61131.7	280	4.58	0.91(0.76-1.08)	925	15.13	0.88(0.80-0.97)	280	4.58	0.96(0.80-1.15)	925	15.13	0.91(0.82-1.00)
Quartile 3	6713	42865.3	183	4.27	0.94(0.77-1.16)	595	13.88	0.88(0.78-0.99)	183	4.27	1.04(0.83-1.30)	595	13.88	0.92(0.81-1.04)
Quartile 4	4848	30667.4	83	2.71	0.75(0.57-0.98)	321	10.47	0.79(0.69-0.91)	83	2.71	0.85(0.63-1.15)	321	10.47	0.85(0.73-1.00)
P for trend					0.085			0.002			0.579			0.065
**Vitamin C**														
Quartile 1	7193	45087.9	208	4.61	1	704	15.61	1	208	4.61	1	704	15.61	1
Quartile 2	6755	43953.9	209	4.75	0.94(0.77-1.14)	668	15.20	0.89(0.80-0.99)	209	4.75	0.97(0.79-1.19)	668	15.20	0.91(0.82-1.02)
Quartile 3	6756	43768.5	193	4.41	0.80(0.66-0.98)	699	15.97	0.86(0.77-0.96)	193	4.41	0.83(0.67-1.03)	699	15.97	0.89(0.80-1.00)
Quartile 4	6751	45428.9	195	4.29	0.97(0.79-1.19)	609	13.41	0.86(0.77-0.97)	195	4.29	1.03(0.81-1.31)	609	13.41	0.92(0.80-1.05)
P for trend					0.425			0.009			0.78			0.177
**Vitamin E**														
Quartile 1	9296	63629.0	403	6.33	1	1262	19.83	1	403	6.33	1	1262	19.83	1
Quartile 2	8012	52944.1	215	4.06	0.81(0.68-0.97)	725	13.69	0.83(0.75-0.92)	215	4.06	0.83(0.69-1.00)	725	13.69	0.84(0.76-0.93)

Quartile 3	5214	32794.7	121	3.69	0.84(0.67-1.06)	412	12.56	0.84(0.74-0.96)	121	3.69	0.88(0.69-1.13)	412	12.56	0.86(0.75-0.98)
Quartile 4	4933	28871.5	66	2.29	0.60(0.45-0.81)	281	9.73	0.73(0.63-0.84)	66	2.29	0.64(0.46-0.87)	281	9.73	0.74(0.63-0.87)
P for trend					0.001			0.001			0.012			0.001
**Folate equivalent**														
Quartile 1	6874	43840.0	266	6.07	1	849	19.37	1	266	6.07	1	849	19.37	1
Quartile 2	6864	44388.4	209	4.71	0.82(0.68-0.99)	693	15.61	0.83(0.75-0.92)	209	4.71	0.82(0.68-0.99)	693	15.61	0.84(0.75-0.93)
Quartile 3	6861	44751.8	183	4.09	0.83(0.68-1.02)	608	13.59	0.82(0.73-0.91)	183	4.09	0.84(0.68-1.04)	608	13.59	0.83(0.74-0.93)
Quartile 4	6856	45259.0	147	3.25	0.74(0.59-0.92)	530	11.71	0.77(0.68-0.87)	147	3.25	0.78(0.61-0.99)	530	11.71	0.80(0.70-0.91)
P for trend					0.009			0.001			0.053			0.001

Model 1: adjustments included age, sex, ethnicity, income, education level, regular exercise, smoking and drinking status, BMI; prevalence of diabetes, hypertension, hyperlipidemia; history of cancer, stroke, cardiovascular disease; medicine control for cholesterol, serum glucose, and blood pressure; dietary nutrient supplement use, AHEI, total daily energy intake per kg and total dietary vitamin intake.

Model 2: all adjustments in Model 1, and the amount of dietary vitamin intake at different time

Supplementary table 5. Modification effect of sex on the association of the dietary VB2, VB6, VC, VE, and Folate equivalent intake in the morning and evening with all-cause and CVD mortality

**Intake time**	**Vitamins**	**CVD Mortality**		**All-cause Mortality**	
		**HR (95% CI)**	***P interaction***	**HR (95% CI)**	***P interaction***
Morning					
	Vitamin B2	0.93(0.81-1.06)	0.278	0.99(0.92-1.07)	0.767
	Vitamin B6	1.00(0.87-1.15)	0.984	1.02(0.95-1.10)	0.613
	Vitamin C	1.03(0.91-1.16)	0.642	1.00(0.93-1.07)	0.921
	Vitamin E	1.06(0.91-1.23)	0.463	0.99(0.91-1.07)	0.791
	Folate equivalent	0.95(0.83-1.08)	0.413	1.02(0.95-1.09)	0.685
Evening					
	Vitamin B2	0.93(0.81-1.07)	0.306	1.03(0.95-1.11)	0.501
	Vitamin B6	0.88(0.76-1.01)	0.075	0.99(0.91-1.07)	0.708
	Vitamin C	0.90(0.80-1.02)	0.100	1.00(0.93-1.07)	0.885
	Vitamin E	0.85(0.73-0.99)	0.041	0.96(0.88-1.04)	0.285
	Folate equivalent	0.96(0.85-1.10)	0.583	1.00(0.93-1.08)	0.940

Adjustments included age, sex, ethnicity, income, education level, regular exercise, smoking and drinking status, BMI; prevalence of diabetes, hypertension, hyperlipidemia; history of cancer, stroke, cardiovascular disease; medicine control for cholesterol, serum glucose, and blood pressure; dietary nutrient supplement use, AHEI, total daily energy intake per kg and total dietary vitamin intake.

Supplementary table 6. Analysis of CVD mortality in term of dietary VE intake quartiles in the evening on participants in Male and Female.

	**Male**		**Female**	
	**Case/N**	**HR (95% CI)**	**Case/N**	**HR (95% CI)**
VE				
Quartile 1	131/3024	1	98/3211	1
Quartile 2	178/4630	1.09(0.87-1.37)	120/4806	0.84(0.64-1.10)
Quartile 3	112/3381	1.08(0.83-1.41)	83/3759	0.75(0.55-1.02)
Quartile 4	52/2324	1.01(0.71-1.43)	31/2320	0.56(0.36-0.87)
P for trend		0.945		0.006

Adjustments included age, ethnicity, income, education level, regular exercise, smoking and drinking status, BMI; prevalence of diabetes, hypertension, hyperlipidemia; history of cancer, stroke, cardiovascular disease; medicine control for cholesterol, serum glucose, and blood pressure; dietary nutrient supplement use, AHEI, total daily energy intake per kg and total dietary vitamin intake.

Supplementary table 7. Modification effect of Hypertension, Hyperlipidemia, Diabetes, Stroke, Cardiovascular disease, and Cancer on the association of the dietary VB2, VB6, VC, VE, and Folate equivalent intake in the morning and evening with all-cause and CVD mortality

**Intake time**	**Diseases**	**Vitamins**	**CVD Mortality**		**All-cause Mortality**	
			**HR (95% CI)**	***P interaction***	**HR (95% CI)**	***P interaction***
Morning	Hypertension					
		Vitamin B2	1.06(0.90-1.24)	0.495	1.04(0.96-1.12)	0.376
		Vitamin B6	1.03(0.88-1.21)	0.710	0.99(0.92-1.08)	0.862
		Vitamin C	0.99(0.85-1.14)	0.833	1.00(0.93-1.07)	0.960
		Vitamin E	1.00(0.84-1.19)	0.956	1.00(0.92-1.09)	0.964
		Folate equivalent	1.01(0.87-1.17)	0.908	1.00(0.93-1.08)	0.929
	Hyperlipidemia					
		Vitamin B2	1.03(0.90-1.17)	0.715	1.01(0.94-1.09)	0.739
		Vitamin B6	1.02(0.89-1.16)	0.828	1.01(0.93-1.08)	0.901
		Vitamin C	1.00(0.88-1.12)	0.949	0.98(0.91-1.04)	0.462
		Vitamin E	1.04(0.90-1.21)	0.572	1.03(0.95-1.12)	0.444
		Folate equivalent	1.05(0.92-1.19)	0.490	1.02(0.95-1.09)	0.617
	Diabetes					
		Vitamin B2	1.00(0.98-1.02)	0.744	1.00(0.99-1.01)	0.377
		Vitamin B6	1.01(0.99-1.02)	0.591	1.00(0.99-1.01)	0.909
		Vitamin C	1.00(0.98-1.02)	0.936	1.01(0.99-1.02)	0.138
		Vitamin E	1.00(0.97-1.03)	0.961	1.00(0.99-1.01)	0.983
		Folate equivalent	1.00(0.99-1.02)	0.714	1.00(0.99-1.01)	0.773
	Stroke					
		Vitamin B2	1.22(1.06-1.40)	0.005	1.02(0.98-1.07)	0.383
		Vitamin B6	1.26(1.10-1.44)	0.001	1.03(0.98-1.08)	0.242
		Vitamin C	1.13(0.99-1.28)	0.073	1.03(0.99-1.08)	0.142
		Vitamin E	1.18(1.00-1.34)	0.050	1.02(0.97-1.07)	0.468
		Folate equivalent	1.39(1.23-1.57)	0.001	1.02(0.98-1.06)	0.414
	Cardiovascular disease					
		Vitamin B2	0.97(0.90-1.05)	0.424	1.01(0.98-1.05)	0.496
		Vitamin B6	0.97(0.89-1.04)	0.376	1.01(0.97-1.04)	0.714
		Vitamin C	1.05(0.98-1.12)	0.178	1.04(1.01-1.07)	0.017
		Vitamin E	0.98(0.90-1.08)	0.714	1.01(0.97-1.05)	0.804

		Folate equivalent	1.00(0.93-1.08)	0.939	1.00(0.97-1.03)	0.983
	Cancer					
		Vitamin B2	1.03(0.87-1.21)	0.767	0.92(0.84-1.00)	0.056
		Vitamin B6	1.07(0.91-1.27)	0.414	0.95(0.87-1.03)	0.212
		Vitamin C	1.01(0.87-1.17)	0.875	0.98(0.91-1.06)	0.639
		Vitamin E	1.02(0.85-1.22)	0.865	0.93(0.84-1.02)	0.132
		Folate equivalent	1.01(0.87-1.19)	0.862	0.96(0.88-1.04)	0.303
Evening	Hypertension					
		Vitamin B2	1.04(0.89-1.23)	0.604	1.01(0.93-1.10)	0.761
		Vitamin B6	0.94(0.80-1.10)	0.428	0.98(0.91-1.07)	0.673
		Vitamin C	1.02(0.88-1.18)	0.797	0.99(0.92-1.06)	0.737
		Vitamin E	0.95(0.80-1.13)	0.575	0.96(0.88-1.04)	0.332
		Folate equivalent	0.97(0.84-1.13)	0.707	0.97(0.90-1.05)	0.458
	Hyperlipidemia					
		Vitamin B2	1.03(0.90-1.18)	0.638	1.02(0.95-1.10)	0.590
		Vitamin B6	1.04(0.90-1.19)	0.596	1.03(0.96-1.11)	0.430
		Vitamin C	1.01(0.90-1.14)	0.852	1.00(0.93-1.07)	0.960
		Vitamin E	1.01(0.87-1.16)	0.942	0.99(0.92-1.08)	0.866
		Folate equivalent	0.88(0.78-1.01)	0.055	0.97(0.91-1.04)	0.430
	Diabetes					
		Vitamin B2	1.00(0.99-1.01)	0.964	1.00(0.99-1.01)	0.741
		Vitamin B6	1.00(0.99-1.01)	0.921	1.00(0.99-1.01)	0.764
		Vitamin C	1.01(0.99-1.03)	0.564	1.00(0.99-1.01)	0.380
		Vitamin E	1.00(0.99-1.02)	0.885	1.00(0.99-1.01)	0.755
		Folate equivalent	1.00(0.99-1.01)	0.960	1.00(0.99-1.01)	0.921
	Stroke					
		Vitamin B2	0.78(0.68-0.91)	0.001	0.96(0.92-1.01)	0.125
		Vitamin B6	0.84(0.72-0.98)	0.024	1.01(0.97-1.06)	0.610
		Vitamin C	0.91(0.79-1.05)	0.202	0.99(0.95-1.04)	0.776
		Vitamin E	0.95(0.80-1.14)	0.606	1.02(0.97-1.07)	0.539
		Folate equivalent	0.88(0.76-1.03)	0.101	1.02(0.98-1.07)	0.330
	Cardiovascular disease					
		Vitamin B2	0.91(0.84-1.00)	0.040	0.98(0.95-1.02)	0.268
		Vitamin B6	0.90(0.82-1.00)	0.046	1.00(0.96-1.04)	0.972
		Vitamin C	1.00(0.93-1.08)	0.958	1.01(0.97-1.04)	0.742

	Vitamin E	0.99(0.91-1.08)	0.884	1.02(0.98-1.06)	0.377
	Folate equivalent	0.98(0.90-1.05)	0.512	1.03(0.99-1.06)	0.113
Cancer					
	Vitamin B2	1.07(0.91-1.27)	0.408	0.98(0.89-1.07)	0.624
	Vitamin B6	1.08(0.91-1.28)	0.358	0.95(0.87-1.04)	0.247
	Vitamin C	1.02(0.88-1.18)	0.814	0.99(0.91-1.07)	0.727
	Vitamin E	1.00(0.83-1.19)	0.967	0.92(0.84-1.02)	0.100
	Folate equivalent	0.99(0.85-1.15)	0.884	0.96(0.89-1.04)	0.340

Adjustments included age, sex, ethnicity, income, education level, regular exercise, smoking and drinking status, BMI; prevalence of diabetes, hypertension, hyperlipidemia; history of cancer, stroke, cardiovascular disease; medicine control for cholesterol, serum glucose, and blood pressure; dietary nutrient supplement use, AHEI, total daily energy intake per kg and total dietary vitamin intake.

Supplementary table 8. Analysis of CVD mortality in term of dietary VB2, VB6, and Folate equivalent intake quartiles in the morning on participants in Stroke group.

	**Stroke group**	
	**Case/N**	**HR (95% CI)**
VB2		
Quartile 1	34/263	1
Quartile 2	19/262	0.53(0.30-0.95)
Quartile 3	38/259	0.89(0.54-1.48)
Quartile 4	38/252	0.76(0.45-1.27)
P for trend		0.714
VB6		
Quartile 1	30/268	1
Quartile 2	32/261	0.94(0.56-1.56)
Quartile 3	30/258	0.72(0.42-1.23)
Quartile 4	37/249	0.83(0.48-1.42)
P for trend		0.465
Folate equivalent		
Quartile 1	33/261	1
Quartile 2	26/260	0.76(0.44-1.30)
Quartile 3	39/257	0.91(0.54-1.55)
Quartile 4	31/258	0.63(0.36-1.09)
P for trend		0.888

Adjustments included age, sex, ethnicity, income, education level, regular exercise, smoking and drinking status, BMI; prevalence of diabetes, hypertension, hyperlipidemia; history of cancer, cardiovascular disease; medicine control for cholesterol, serum glucose, and blood pressure; dietary nutrient supplement use, AHEI, total daily energy intake per kg and total dietary vitamin intake.

Supplementary table 9. Analysis of CVD mortality in term of dietary VB2, and VB6 equivalent intake quartiles in the evening on participants in Stroke group.

	**Stroke group**	
	**Case/N**	**HR (95% CI)**
VB2		
Quartile 1	37/259	1
Quartile 2	27/264	0.63(0.37-1.05)
Quartile 3	32/265	0.87(0.52-1.45)
Quartile 4	33/248	1.08(0.63-1.82)
P for trend		0.726
VB6		
Quartile 1	27/252	1
Quartile 2	43/275	1.49(0.90-2.47)
Quartile 3	33/260	1.45(0.85-2.50)
Quartile 4	26/249	1.09(0.59-1.99)
P for trend		0.782

Adjustments included age, sex, ethnicity, income, education level, regular exercise, smoking and drinking status, BMI; prevalence of diabetes, hypertension, hyperlipidemia; history of cancer, cardiovascular disease; medicine control for cholesterol, serum glucose, and blood pressure; dietary nutrient supplement use, AHEI, total daily energy intake per kg and total dietary vitamin intake.

Supplementary table 10. Analysis of CVD mortality in term of dietary VB2 and VB6 intake quartiles in the evening on participants in Cardiovascular disease group.

	**Cardiovascular disease group**	
	**Case/N**	**HR (95% CI)**
VB2		
Quartile 1	34/283	1
Quartile 2	34/305	0.97(0.60-1.58)
Quartile 3	43/298	1.40(0.87-2.26)
Quartile 4	39/278	1.35(0.80-2.26)
P for trend		0.083
VB6		
Quartile 1	37/287	1
Quartile 2	36/302	1.00(0.63-1.60)
Quartile 3	42/296	1.41(0.87-2.28)
Quartile 4	35/279	1.39(0.83-2.33)
P for trend		0.139

Adjustments included age, sex, ethnicity, income, education level, regular exercise, smoking and drinking status, BMI; prevalence of diabetes, hypertension, hyperlipidemia; history of cancer, stroke; medicine control for cholesterol, serum glucose, and blood pressure; dietary nutrient supplement use, AHEI, total daily energy intake per kg and total dietary vitamin intake.
