# Supplementary figures and images for: The Association of Dietary Vitamin Intake Time Across a Day With Cardiovascular Disease and All-Cause Mortality
Source: Front Cardiovasc Med. 2022 Mar 23;9:822209. doi: 10.3389/fcvm.2022.822209 (PMC8984283; doi:10.3389/fcvm.2022.822209)

a

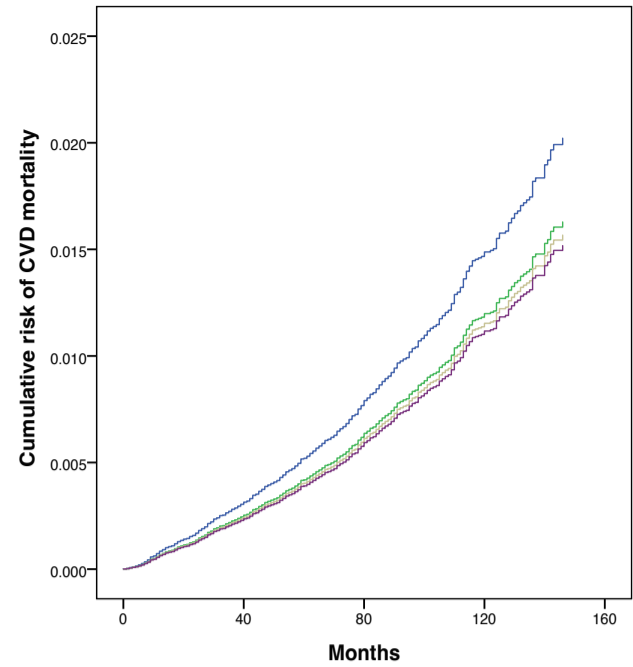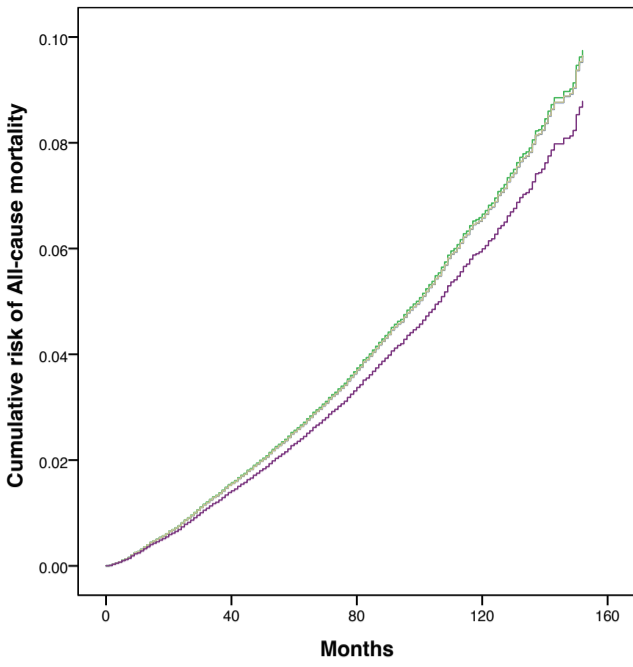

b

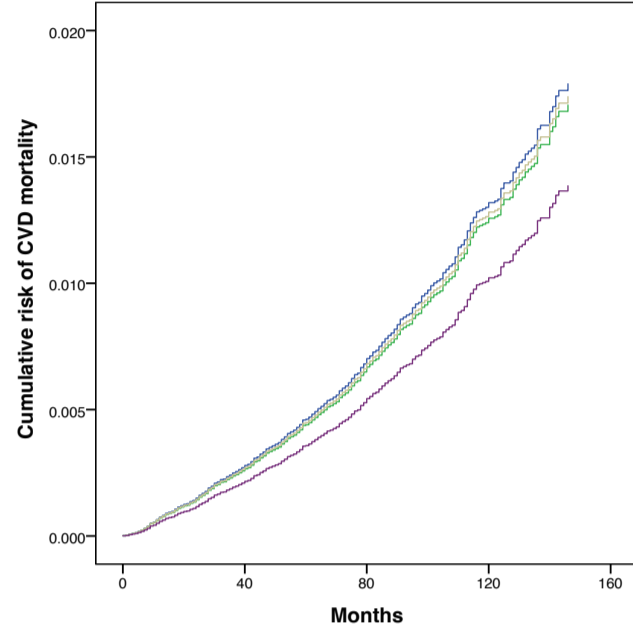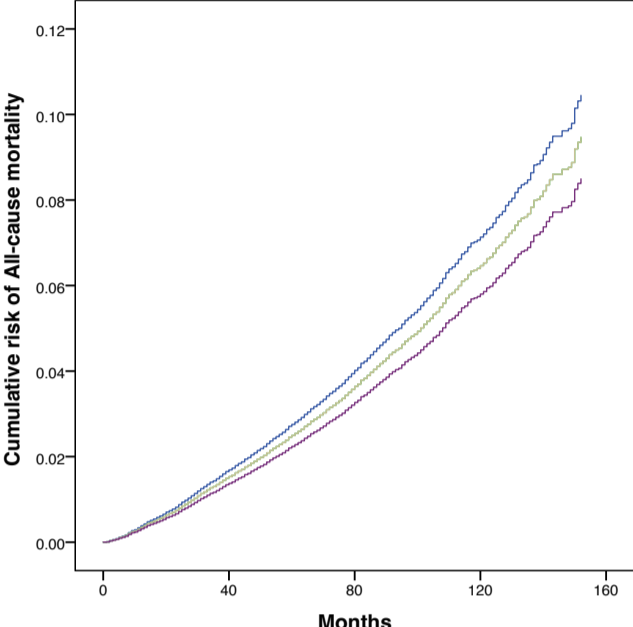

c

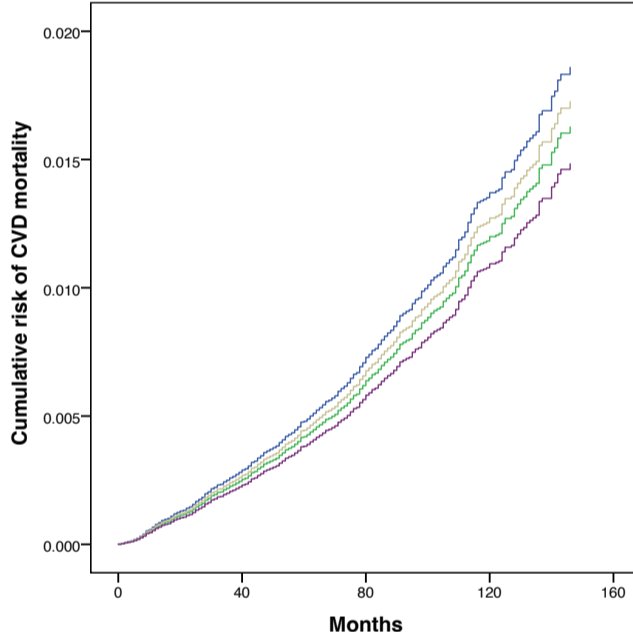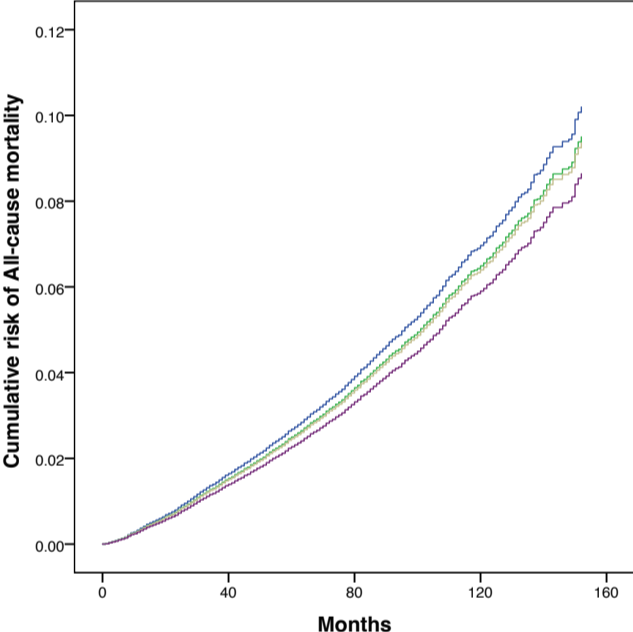

d

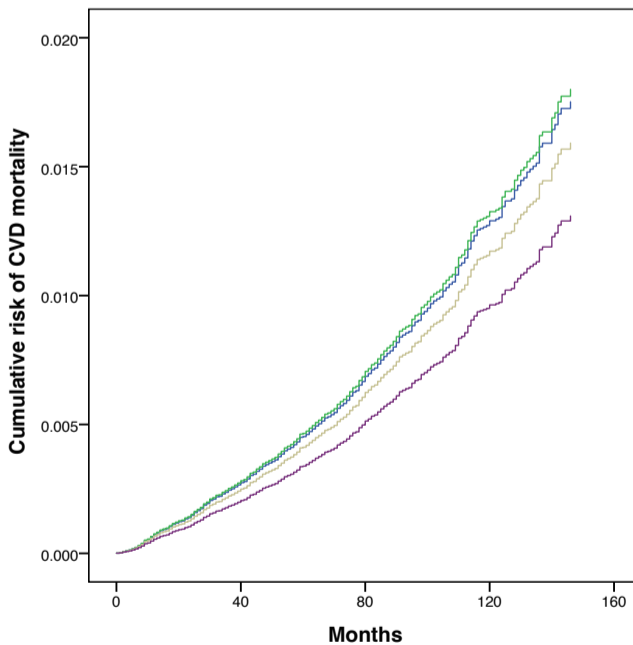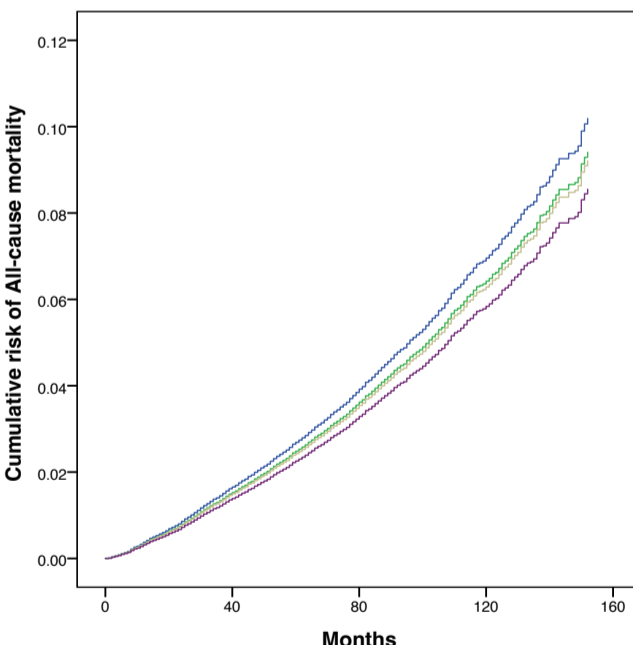

e

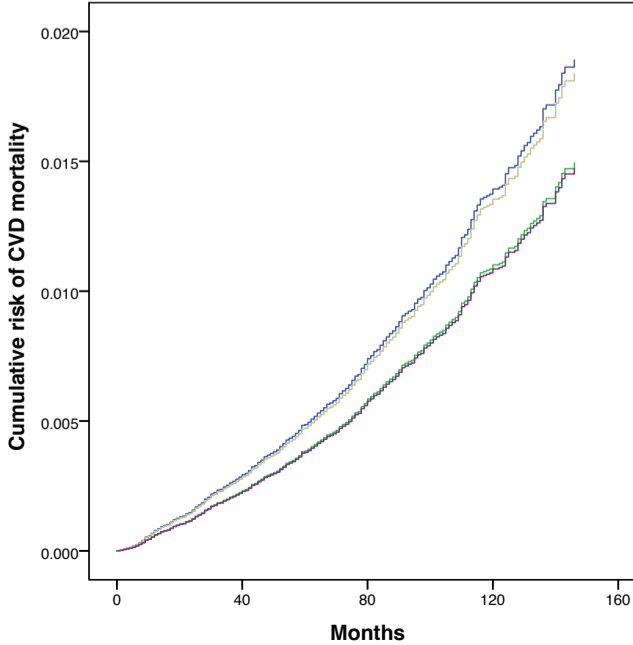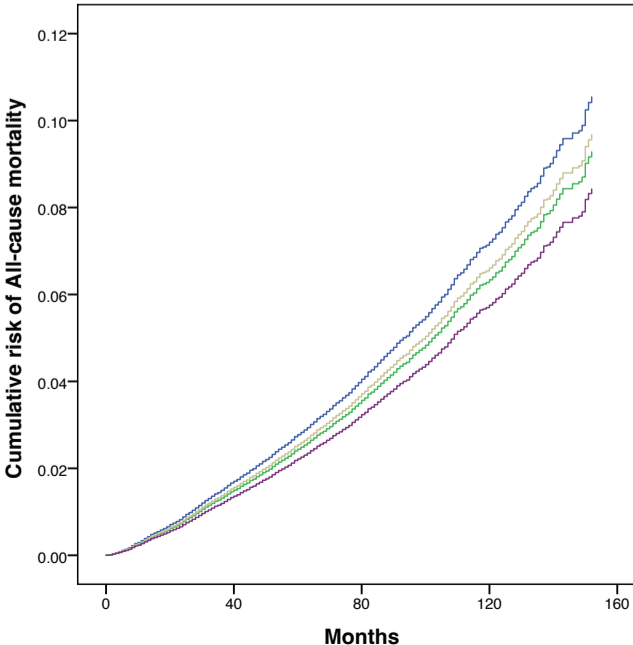

Supplement: Supplementary file 2 [file Image_1.pdf]
